# Effectiveness of Whole, Inactivated, Low Pathogenicity Influenza A(H7N9) Vaccine against Antigenically Distinct, Highly Pathogenic H7N9 Virus

**DOI:** 10.3201/eid2410.180403

**Published:** 2018-10

**Authors:** Masato Hatta, Gongxun Zhong, Shiho Chiba, Tiago J.S. Lopes, Gabriele Neumann, Yoshihiro Kawaoka

**Affiliations:** University of Wisconsin–Madison, Madison, Wisconsin, USA (M. Hatta, G. Zhong, S. Chiba, T.J.S. Lopes, G. Neumann, Y. Kawaoka);; University of Tokyo, Tokyo, Japan (Y. Kawaoka)

**Keywords:** influenza, H7N9, highly pathogenic, vaccine, viruses, low pathogenicity

## Abstract

The recent emergence of highly pathogenic influenza A(H7N9) variants poses a great risk to humans. We show that ferrets vaccinated with low pathogenicity H7N9 virus vaccine do not develop severe symptoms after infection with an antigenically distinct, highly pathogenic H7N9 virus. These results demonstrate the protective benefits of this H7N9 vaccine.

Low pathogenicity influenza A(H7N9) viruses, which cause mild or asymptomatic disease in poultry, have caused >1,564 human infections since March 2013, with a case-fatality rate of ≈40% (*1*–*5*). Recently, highly pathogenic H7N9 viruses, characterized by multiple basic amino acids at the cleavage site of their hemagglutinin (HA) protein, have emerged. More than 750 cases of human H7N9 infections in 2017 (*6*) and the emergence of highly pathogenic H7N9 viruses emphasize the need for effective vaccines against low pathogenicity and highly pathogenic H7N9 viruses. We examined whether a World Health Organization (WHO) candidate vaccine based on a low pathogenicity H7N9 influenza virus would protect ferrets against an antigenically distinct, highly pathogenic H7N9 influenza virus.

## The Study

We generated a recombinant virus (HK125–HYPR8) that possesses the HA and neuraminidase (NA) genes of a low pathogenicity WHO-recommended H7N9 candidate vaccine virus (A/Hong Kong/125/2017 [*7*]) and the remaining genes from a high-yield A/Puerto Rico/8/34 (PR8) vaccine backbone virus (*8*). The HK125–HYPR8 virus was inactivated with β-propiolactone and purified through sucrose gradient ultracentrifugation.

**Figure 1:**
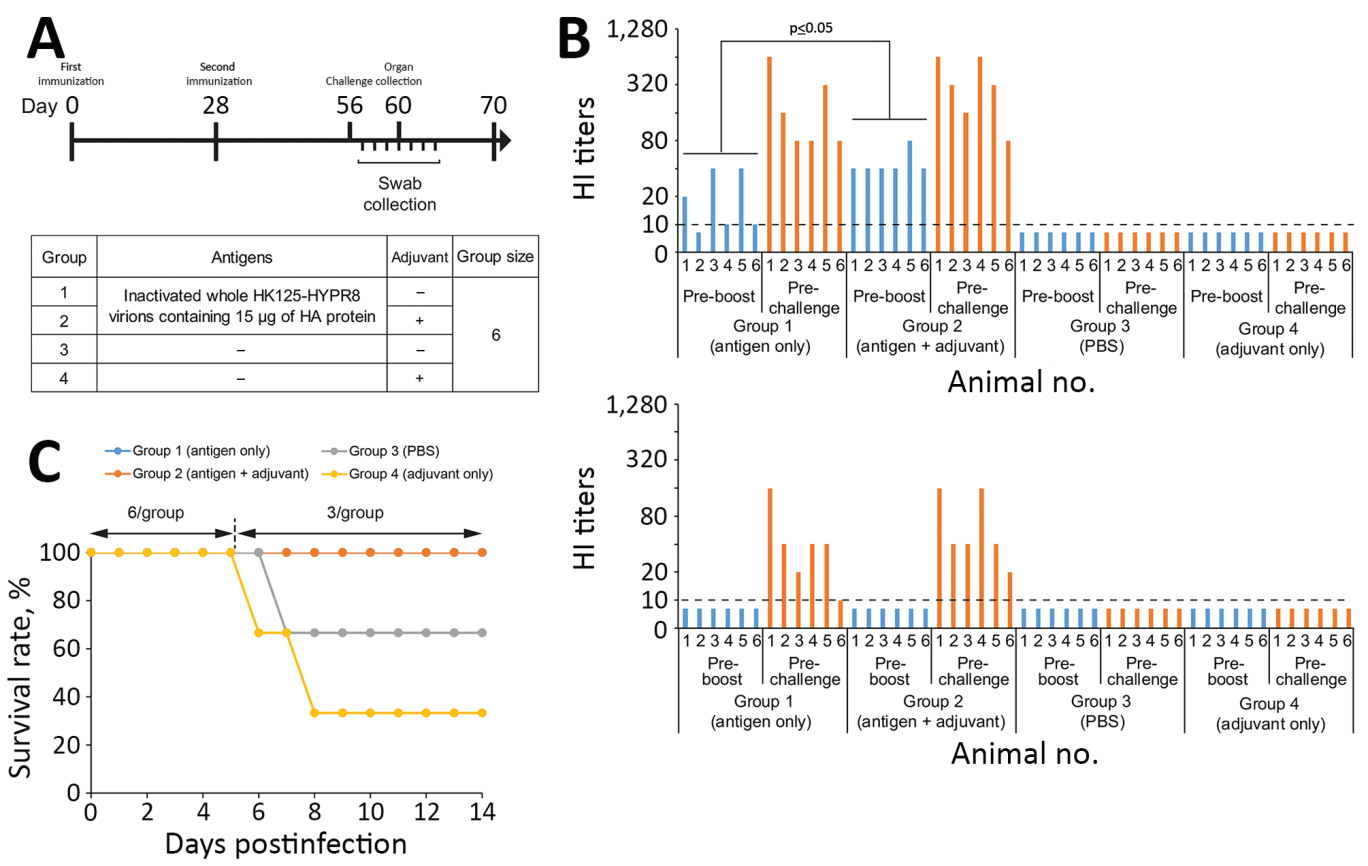
Study design, HI titers after vaccination, and survival rates of vaccinated and nonvaccinated ferrets challenged with highly pathogenic influenza A(H7N9) virus. A) Study design. Six ferrets per group were immunized with inactivated whole HK125–HYPR8 virions containing 15 μg of HA protein without (group 1) or with adjuvant (group 2); control animals were vaccinated with PBS (group 3) or adjuvant (group 4). Animals were vaccinated intramuscularly twice 28 days apart. Twenty-eight days after the second immunization, ferrets were challenged with highly pathogenic H7N9 rGD/3-NA294R virus. Throat and nasal swab specimens were collected on days 1–7 postchallenge; 3 animals per group were euthanized on day 4 postchallenge to assess virus titers in organs. B) HI titers after vaccination. HI assays were performed against HK125–HYPR8 (upper panel) and rGD/3-NA294R (lower panel) with ferret sera collected before the second immunization (preboost) and before challenge (prechallenge). Statistical significance was determined as described in the Technical Appendix. C) Survival rates. Survival was monitored for 14 days after challenge. Because 3 ferrets were euthanized on day 4 postchallenge for organ sampling, the survival rate was calculated on the basis of a group size of n = 3 thereafter. HA, hemagglutinin; HI, hemagglutination inhibition; PBS, phosphate-buffered saline.

We vaccinated 5-month-old female ferrets (6 per group) that were serologically negative for currently circulating human influenza viruses with 15 μg of HA of inactivated whole HK125–HYPR8 virions without adjuvant (Group 1) or mixed at a 1:1 ratio with AddaVax adjuvant (InvivoGen, San Diego, CA, USA), a squalene-based oil-in-water nanoemulsion similar to MF59 (*9*) (group 2); control animals received phosphate-buffered saline (group 3) or adjuvant (group 4) (Figure 1, panel A). All animals were vaccinated intramuscularly in both hind legs twice, 28 days apart.

Twenty-eight days after the second immunization, we intranasally challenged ferrets with 10^6^ PFUs of highly pathogenic H7N9 rGD/3-NA294R virus (a neuraminidase inhibitor–sensitive subpopulation of highly pathogenic A/Guangdong/17SF003/2016 H7N9 virus) (*10*). These vaccine and challenge viruses belong to the Yangtze River Delta lineage of H7N9 viruses, which is responsible for recent infections of humans with highly pathogenic H7N9 viruses (*6*). However, A/Hong Kong/125/2017 and the A/Guangdong/17SF003/2016 challenge virus differ antigenically (*11*) (Technical Appendix Table 1).

We monitored clinical signs, body weight, and body temperature daily for 14 days and collected throat and nasal swab specimens every day until day 7 postchallenge. On day 4 postchallenge, we euthanized 3 ferrets from each group and collected organs (lung, trachea, nasal turbinates, olfactory bulbs, and brain tissues pooled from anterior and posterior brain sections) for virus titration. We conducted statistical analysis of hemagglutinin inhibition (HI) titers, virus titers in swab and organ samples, and bodyweight and temperature changes among groups (Technical Appendix Tables 2–21). We defined statistical significance as p<0.05.

After 1 immunization, HI titers were significantly lower in the ferrets immunized with nonadjuvanted HK125–HYPR8 vaccine than in those immunized with AddaVax-adjuvanted HK125–HYPR8 vaccine (p = 0.038; Figure 1, panel B; Technical Appendix Table 2); however, after 2 immunizations, ferrets vaccinated with or without adjuvant (groups 1 and 2) developed high HI titers against HK125–HYPR8 virus. Vaccination with HK125–PR8 vaccine did not elicit measurable HI titers against the rGD/3-NA294R challenge virus after the first immunization but elicited reasonably high titers after the second immunization (Figure 1, panel B). After challenge with highly pathogenic H7N9 virus, nonvaccinated ferrets (groups 3 and 4) became lethargic, experienced diarrhea, and lost appetite and bodyweight on days 2–6 postinfection (Technical Appendix Figure), whereas vaccinated ferrets showed no noticeable symptoms. In addition, nonvaccinated ferrets demonstrated statistically higher body temperature than vaccinated ferrets on days 1, 2, 3, 5, and 6 postchallenge (Technical Appendix Figure, Table 5). One ferret in group 3 and 2 ferrets in group 4 had to be euthanized on days 6–8 postinfection (Figure 1, panel C) because of severe symptoms (neurologic signs or inability to remain upright). In contrast, none of the vaccinated ferrets had any symptoms, indicating a protective effect of the low pathogenicity H7N9 vaccine against the challenge virus.

**Figure 2:**
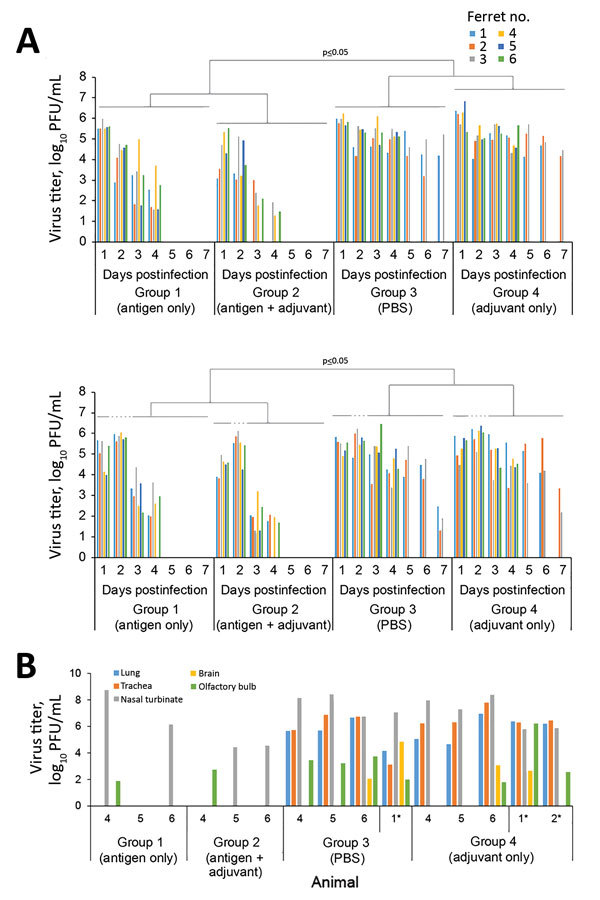
Virus titers in throat and nasal swab specimens and in the organs of vaccinated and nonvaccinated ferrets challenged with highly pathogenic influenza A(H7N9) virus. A) Virus titers in swab samples. Throat and nasal swabs were collected on days 1–7 postchallenge. Virus titers were determined based on plaque assays in MDCK cells. Statistical significance was determined as described in the Technical Appendix. B) Three ferrets from each group were euthanized on day 4 postchallenge for virus titration in the indicated organs. We also assessed virus titers in organs of ferrets that were euthanized because of severe symptoms (*). Virus titers were determined based on plaque assays in MDCK cells. Numbers along baseline indicate animal number. PBS, phosphate-buffered saline.

Analysis of throat and nasal swab samples demonstrated replication of highly pathogenic challenge virus in all ferrets (Figure 2, panel A). However, virus titers started to decline in vaccinated ferrets by day 3 postchallenge, and the infection was resolved by day 5 postchallenge; in contrast, nonvaccinated ferrets continued to shed high titers of challenge virus 4–7 days postchallenge. The virus titers in nasal swab samples on days 1, 3, 4, 5, 6, and 7 postchallenge and those in throat swab samples on days 1–7 postchallenge from nonvaccinated ferrets were significantly higher than those in vaccinated ferrets (Technical Appendix Table 10). Thus, vaccination with HK125–HYPR8 virus led to reduced replication of the challenge virus in the upper respiratory tract of infected ferrets.

On day 4 postinfection, we euthanized 3 animals per group and determined virus titers in organs. We also assessed virus titers in organs of ferrets that were euthanized because of severe disease symptoms. In nonvaccinated ferrets, we detected high titers of virus in respiratory organs; in addition, we recovered virus from the olfactory bulbs or pooled samples from anterior and posterior sections of the brains of 7 of the 9 animals tested (Figure 2, panel B). In vaccinated ferrets, we detected virus in the nasal turbinates of 4 of 6 animals and in the olfactory bulbs of 2 of 6 animals. We recovered no virus from the tracheas, lungs, or pooled samples from anterior and posterior brain sections (Figure 2, panel B), indicating that vaccination with HK125–HYPR8 prevented challenge virus replication in the lower respiratory organs.

## Conclusions

We report the effectiveness of a whole, inactivated, low pathogenicity H7N9 vaccine against an antigenically distinct, highly pathogenic H7N9 virus in a ferret model. Vaccination prevented challenge virus replication in the lower respiratory organs, led to faster virus clearance in the upper respiratory organs, and prevented severe disease and death in ferrets, although the HI titers against the rGD/3-NA294R challenge virus were lower than those against the HK125–HYPR8 vaccine virus. Statistical analyses demonstrated that HI titers against the HK125–HYPR8 vaccine virus after the first immunization were significantly higher (p = 0.038) in animals immunized with adjuvanted vaccine compared with animals immunized with nonadjuvanted vaccine (Figure 1, panel B; Technical Appendix Table 2). Bodyweight changes after challenge were significantly milder (p = 0.0132–0.0489 on days 4–10, 12, and 13) in ferrets immunized with adjuvanted vaccine than in those vaccinated with nonadjuvanted vaccine. In addition, virus titers in nasal swabs on days 3 and 4 postchallenge (p = 0.0052 on day 3; p = 0.0163 on day 4) and in throat swabs on days 1, 3, and 4 (p = 0.0047 on day 1; p = 0.0003 on days 3 and 4) in ferrets immunized with nonadjuvanted vaccine were significantly higher than in those ferrets immunized with adjuvanted vaccine (Technical Appendix Tables 9, 11), suggesting superior efficacy with Addavax.

Previously, WHO selected several low pathogenicity H7N9 candidate vaccine viruses, including A/Hong Kong/125/2017 (*7*). With the emergence of highly pathogenic H7N9 viruses that are antigenically distinct from previously circulating H7N9 viruses, WHO has updated its recommendations, and a candidate vaccine virus for highly pathogenic H7N9 viruses is now available (*12*). We tested whether in the event of a large-scale outbreak of highly pathogenic H7N9 viruses, candidate vaccine viruses to antigenically distinct H7N9 viruses might serve as a first line of defense. Our results in ferrets indicate the potential of a whole, inactivated vaccine based on a low pathogenicity H7N9 virus to prevent severe disease with fatal outcome after infection with an antigenically distinct, highly pathogenic H7N9 virus.

Technical Appendix Additional information from the study of low pathogenicity influenza A(H7N9) vaccine against highly pathogenic H7N9.
