# Supplementary material for: Effectiveness of Whole, Inactivated, Low Pathogenicity Influenza A(H7N9) Vaccine against Antigenically Distinct, Highly Pathogenic H7N9 Virus
Source: Emerg Infect Dis. 2018 Oct;24(10):1910–3. doi: 10.3201/eid2410.180403 (PMC6154142; doi:10.3201/eid2410.180403)
Supplement: Technical Appendix — Additional information from the study of low pathogenicity influenza A(H7N9) vaccine against highly pathogenic H7N9. [file 18-0403-Techapp-s1.pdf]

Article DOI: https://doi.org/10.3201/eid2410.180403

Whole, Inactivated, Low Pathogenicity Influenza A(H7N9) Vaccine against Antigenically Distinct, Highly Pathogenic H7N9

Technical Appendix

Supplementary Methods

Cells

Madin-Darby canine kidney (MDCK) cells (obtained from ATCC) were maintained in Eagle's minimal essential medium (MEM) containing 5% newborn calf serum and antibiotics. Human embryonic kidney 293T cells (obtained from ATCC) were propagated in Dulbecco's modified Eagle's medium (DMEM) containing 10% fetal calf serum (FCS) with antibiotics. All cells were maintained at 37°C with 5% CO_2 unless otherwise stated.

Virus and Reverse Genetics

The sequences of the haemagglutinin (HA) and neuraminidase (NA) genes of a low pathogenic WHO-recommended H7N9 candidate vaccine virus (A/Hong Kong/125/2017, H7N9) (*1*) were obtained from GenBank (accession numbers: CY235363 and CY235364, respectively). Based on the obtained sequences, the HA and NA genes were oligo-synthesized by SGI-DNA (La Jolla, CA) and cloned into a plasmid for viral RNA production (pPolI vector) (*2*). Plasmid-based reverse genetics for generating HK125-HYPR8 virus possessing the HA and NA genes of A/Hong Kong/125/2017 and the remaining genes from our high-yield A/Puerto Rico/8/34 (PR8) vaccine backbone virus was performed as previously described (*2*,*3*). At 48 h post-transfection, culture supernatants were collected and inoculated to MDCK cells for virus propagation. The virus stock was sequenced to confirm the absence of unwanted mutations.

Vaccine Preparation

The HK125-HYPR8 virus was propagated in 10-day-old embryonated chicken eggs. The viruses in the allantoic fluids were inactivated with 0.1% β-propiolactone (final concentration) at 4°C overnight and then purified through ultracentrifugation by using a linear 20%–50% (w/v) sucrose gradient. The HA amount of purified virus was calculated based on the intensities of the viral protein bands separated on a 4%–12% (wt/vol) NuPAGE Bis-Tris gel (Thermo Fisher Scientific) and the amount of total viral proteins was determined by using a Pierce BCA Protein assay kit (Thermo Fisher Scientific).

Animal Experiments

All animal experiments were approved by the Institutional Animal Care and Use Committee (IACUC) at the University of Wisconsin-Madison, which also approved the protocol used (protocol numbers V00806). The facilities where this research was conducted are fully accredited by the Association for the Assessment and Accreditation of Laboratory Animal Care International.

Ferret Vaccine-Challenge Experiment

Five-month-old female ferrets (Triple F Farms), which were serologically negative by hemagglutination inhibition assay for currently circulating human influenza viruses, were used in this study. Six ferrets per group were vaccinated with 15 μg of HA of inactivated whole HK125-HYPR8 virions without adjuvant (Group 1) or mixed at a 1:1 ratio with AddaVax adjuvant (InvivoGen) (Group 2); control animals received PBS (Group 3) or adjuvant (Group 4) (Figure 1, panel A). All animals were vaccinated intramuscularly in both hind legs twice 28 days apart.

Twenty-eight days after the second immunization, ferrets were intranasally challenged with 10^6 PFUs (PFU) of highly pathogenic H7N9 rGD/3-NA294R virus (a neuraminidase inhibitor-sensitive subpopulation of highly pathogenic A/Guangdong/17SF003/2016 H7N9 virus) (*4*). Clinical signs, bodyweight, and body temperature were monitored daily for 14 days. Throat and nasal swabs were collected every day until day 7 post-challenge. On day 4 post-challenge, three ferrets from each group were euthanized and organs (lung, trachea, nasal turbinates, olfactory bulbs, and brain tissues pooled from anterior and posterior brain sections) were collected for virus titration.

Hemagglutination Inhibition (HI) Assay

To detect hemagglutination inhibition (HI) activity (https://www.cdc.gov/flu/professionals/laboratory/antigenic.htm), serum samples were treated with receptor-destroying enzyme (RDE; Denka Seiken Co., Ltd) at 37°C for 16–20 h, followed by RDE inactivation at 56°C for 30–60 min. The treated sera were serially diluted 2-fold with PBS in 96-well U-bottom microtiter plates (Thermo Scientific, Rochester, New York, USA) and mixed with the amount of virus equivalent to eight hemagglutination units, followed by incubation at room temperature (25°C) for 30 min. After 50 μL of 0.5% turkey red blood cells was added to the mixtures, they were gently mixed and incubated at room temperature for a further 45 min. HI titers are expressed as the inverse of the highest antibody dilution that inhibited hemagglutination.

Statistical Analysis

Body temperature, bodyweight, nasal, and throat swabs were analyzed using a linear mixed model, with the groups and time as fixed effects, and the animals as random effects.

The commands lmer, lsmeans, and cld were used for the analysis, and all groups were compared to each other (pairwise). The *p*-values were adjusted using Holm's method. For the comparison of the HI titers, we used two-tailed unpaired t-tests, and adjusted the *p*-values using Holm's method. The virus titers from the organs were analyzed using one-way ANOVA, followed by Tukey's post-hoc test.

Biosafety and Biosecurity

All recombinant DNA protocols were approved by the University of Wisconsin-Madison's Institutional Biosafety Committee after risk assessments were conducted by the Office of Biologic Safety. In addition, the University of Wisconsin-Madison Biosecurity Task Force regularly reviews the research program and ongoing activities of the laboratory. The task force has a diverse skill set and provides support in the areas of biosafety, facilities, compliance, security, and health. Members of the Biosecurity Task Force are in frequent contact with the principal investigator and laboratory personnel to provide oversight and assure biosecurity. All experiments with live highly pathogenic H7N9 virus were performed in biosafety level 3 agricultural (BSL-3Ag) laboratories at the University of Wisconsin-Madison approved for such

use by the Centers for Disease Control and Prevention (CDC) and Animal and Plant Health Inspection Service (APHIS). Staff working in BSL-3Ag wear disposable overalls and powered air-purifying respirators.

The BSL-3Ag facility at University of Wisconsin-Madison was designed to exceed the standards outlined in *Biosafety in Microbiological and Biomedical Laboratories* (5th edition; http://www.cdc.gov/biosafety/publications/bmbl5/BMBL.pdf). Features include controlled access, entry/exit through a shower change room, effluent decontamination, negative air-pressure, double-door autoclaves, gas decontamination ports, HEPA-filtered supply and double-HEPA-filtered exhaust air, double-gasketed watertight and airtight seals, and airtight dampers on all ductwork. The structure is pressure-decay tested regularly. The University of Wisconsin-Madison facility has a dedicated alarm system that monitors all building controls (~500 possible alerts). Redundancies and emergency resources are built into the facility, including two air handlers, two compressors, two filters wherever filters are needed, two effluent sterilization tanks, two power feeds to the building, an emergency generator in case of a power failure, and other physical containment measures in the facility that operate without power. Biosecurity monitoring of the facility is ongoing. All personnel undergo Select Agent security risk assessment by the United States Criminal Justice Information Services Division and complete rigorous biosafety, BSL-3, and Select Agent training before participating in BSL-3-level experiments. Refresher training, including drills and review of emergency plans, is scheduled on a regular basis. The principal investigator participates in training sessions and emphasizes compliance to maintain safe operations and a responsible research environment. The laboratory occupational health plan is in compliance with the University of Wisconsin-Madison Occupational Health Program. Select Agent virus inventory, secured behind two physical barriers, is checked monthly and documentation is submitted to the University of Wisconsin-Madison Select Agent Program Manager. Virus inventory is submitted 1–2 times per year to the file holder in the Division of Select Agents and Toxins at the CDC. The research program, procedures, occupational health plan, documentation, security, and facilities are reviewed annually by the University of Wisconsin-Madison Responsible Official and at regular intervals by the CDC and the APHIS as part of the University of Wisconsin-Madison Select Agent Program.

References

1. World Health Organization (WHO). Summary of status of development and availability of avian influenza A(H7N9) candidate vaccine viruses and potency testing reagents [cited 2017 Sep 28]. http://www.who.int/influenza/vaccines/virus/candidates_reagents/summary_a_h7n9_cvv_20170928.pdf?ua=1

2. Neumann G, Watanabe T, Ito H, Watanabe S, Goto H, Gao P, et al. Generation of influenza A viruses entirely from cloned cDNAs. Proc Natl Acad Sci U S A. 1999;96:9345–50. PubMed http://dx.doi.org/10.1073/pnas.96.16.9345

3. Ping J, Lopes TJ, Nidom CA, Ghedin E, Macken CA, Fitch A, et al. Development of high-yield influenza A virus vaccine viruses. Nat Commun. 2015;6:8148. PubMed http://dx.doi.org/10.1038/ncomms9148

4. Imai M, Watanabe T, Kiso M, Nakajima N, Yamayoshi S, Iwatsuki-Horimoto K, et al. A Highly pathogenic avian H7N9 influenza virus isolated from a human is lethal in some ferrets infected via respiratory droplets. Cell Host Microbe. 2017;22:615–626.e8. PubMed http://dx.doi.org/10.1016/j.chom.2017.09.008

Technical Appendix Table 1. Antigenic differences among H7 viruses by hemagglutination inhibition assays

	Hemagglutination inhibition (HI) titers*							
	Monoclonal antibodies against HA from						Antisera against	
	A/seal/Massachusetts/1/80 (H7N7)			A/Anhui/1/2013 (H7N9)			A/Netherlands/219/03 (H7N7)	HA and NA from A/Hong Kong/125/2017
Virus	46/6	55/3	58/2	2–20–20	3–7-19	19–17–20	NR-9226	3,031
H7N9								
HK125-HYPR8	6,400	12,800	3,200	1,600	1,600	1,600	2,560	**640**
(HA and NA from A/Hong Kong/125/2017)								
A/Guangdong/17SF003/2016	100	3,200	800	400	400	400	40	80
A/Anhui/1/2013	3,200	25,600	3,200	**6,400**	**12,800**	**6,400**	1,280	640
H7N7								
A/seal/Massachusetts/ 1/1980	**6,400**	**12,800**	**3,200**	800	800	800	640	40

*HI titers are described as the inverse of the highest antibody dilution that inhibited hemagglutination. Values obtained with homologous antibodies are shown in bold. Monoclonal antibodies against the HA proteins of A/seal/Massachusetts/1/80 (H7N7) and A/Anhui/1/2013 (H7N9) viruses, and ferret antisera against A/Hong Kong/125/2017 were generated in our laboratory. Goat antiserum against A/Netherlands/219/03 (H7N7) was obtained from BEI Resources.

Technical Appendix Table 2. Statistical analysis of HI titers of groups 1 and 2 against HK125-HYPR8 in Figure 1, panel B (Upper panel).

A	B	Stage	*P* value
Group 1	Group 2	Pre-boost	0.0380
Group 1	Group 2	Pre-challenge	0.3381

The two groups listed in columns A and B were compared.
Cyan: Values in column B are significantly higher than those in column A.

Technical Appendix Table 3. Statistical analysis of HI titers of groups 1 and 2 against rGD/3-NA294R in Figure 1, panel B (Lower panel).

A	B	Stage	*P* value
Group 1	Group 2	Pre-boost	N.A.
Group 1	Group 2	Pre-challenge	0.4871

The two groups listed in columns A and B were compared.
N.A.: not applicable

Technical Appendix Table 4. Statistical analyses of body temperature changes in the Technical Appendix Figure (Comparison of the indicated groups)

A	B	Days post-challenge	Estimate	t-ratio	*P* value
Group 4	Group 2	0	0.1000	0.2670	0.7898
		1	0.4500	1.2015	0.2314
		2	1.1667	3.1151	0.0022
		3	1.3167	3.5156	0.0006
		4	–0.0667	–0.1780	0.8590
		5	1.0333	1.9510	0.0529
		6	0.6667	1.2587	0.2100
		7	–0.4115	–0.6944	0.4885
		8	–0.4777	–0.6366	0.5253
		9	0.7223	0.9624	0.3373
		10	1.5889	2.1173	0.0358
		11	0.6556	0.8736	0.3837
		12	–0.7111	–0.9475	0.3448
		13	0.3556	0.4739	0.6363
		14	–0.2444	–0.3257	0.7451
Group 4	Group 1	0	0.3167	0.8455	0.3991
		1	0.9833	2.6256	0.0095
		2	1.4833	3.9606	0.0001
		3	1.2167	3.2486	0.0014
		4	0.5333	1.4240	0.1565
		5	1.1333	2.1398	0.0339
		6	1.2667	2.3915	0.0180
		7	0.3218	0.5430	0.5879
		8	0.0889	0.1185	0.9058
		9	0.5889	0.7848	0.4338
		10	0.6556	0.8736	0.3837
		11	1.3223	1.7620	0.0800
		12	–0.5111	–0.6810	0.4969
		13	–0.1111	–0.1480	0.8825
		14	–0.1777	–0.2368	0.8131
Group 4	Group 3	0	0.1333	0.3560	0.7223
		1	–0.9333	–2.4921	0.0138
		2	0.7333	1.9581	0.0520
		3	–0.0500	–0.1335	0.8940
		4	–0.5833	–1.5575	0.1214
		5	–1.0333	–1.9510	0.0529
		6	–1.0000	–1.8880	0.0609
		7	–0.8782	–1.4818	0.1404
		8	–0.9495	–1.1937	0.2344
		9	0.2005	0.2520	0.8014
		10	1.1005	1.3834	0.1685
		11	0.9505	1.1949	0.2340
		12	–0.5495	–0.6908	0.4907
		13	–0.4995	–0.6280	0.5309
		14	–0.5495	–0.6908	0.4907
Group 2	Group 1	0	0.2167	0.5785	0.5638
		1	0.5333	1.4240	0.1565
		2	0.3167	0.8455	0.3991
		3	–0.1000	–0.2670	0.7898
		4	0.6000	1.6020	0.1112

A	B	Days post-challenge	Estimate	t-ratio	*P* value
		5	0.1000	0.1888	0.8505
		6	0.6000	1.1328	0.2590
		7	0.7333	1.3846	0.1682
		8	0.5667	1.0699	0.2863
		9	–0.1333	–0.2517	0.8016
		10	–0.9333	–1.7622	0.0800
		11	0.6667	1.2587	0.2100
		12	0.2000	0.3776	0.7062
		13	–0.4667	–0.8811	0.3796
		14	0.0667	0.1259	0.9000
Group 2	Group 3	0	0.0333	0.0890	0.9292
		1	–1.3833	–3.6936	0.0003
		2	–0.4333	–1.1570	0.2490
		3	–1.3667	–3.6491	0.0004
		4	–0.5167	–1.3795	0.1697
		5	–2.0667	–3.9019	0.0001
		6	–1.6667	–3.1467	0.0020
		7	–0.4667	–0.8811	0.3796
		8	–0.4718	–0.7961	0.4272
		9	–0.5218	–0.8805	0.3800
		10	–0.4885	–0.8243	0.4111
		11	0.2949	0.4975	0.6195
		12	0.1615	0.2725	0.7856
		13	–0.8551	–1.4430	0.1510
		14	–0.3051	–0.5149	0.6074
Group 1	Group 3	0	–0.1833	–0.4895	0.6252
		1	–1.9167	–5.1177	0.0000
		2	–0.7500	–2.0026	0.0470
		3	–1.2667	–3.3821	0.0009
		4	–1.1167	–2.9816	0.0033
		5	–2.1667	–4.0907	0.0001
		6	–2.2667	–4.2795	0.0000
		7	–1.2000	–2.2656	0.0249
		8	–1.0385	–1.7523	0.0817
		9	–0.3885	–0.6555	0.5131
		10	0.4449	0.7506	0.4540
		11	–0.3718	–0.6274	0.5313
		12	–0.0385	–0.0649	0.9483
		13	–0.3885	–0.6555	0.5131
		14	–0.3718	–0.6274	0.5313

The two groups listed in columns A and B were compared.
Orange: Values in columns A are significantly higher than those in column B.
Cyan: Values in columns B are significantly higher than those in column A.

Technical Appendix Table 5. Statistical analyses of body temperature changes in the Technical Appendix Figure [Comparison of vaccinated (groups 1 and 2 merged) and unvaccinated (groups 3 and 4 merged) groups].

A	B	Days post-challenge	Estimate	t-ratio	*P* value
Non-vaccinated	Vaccinated	0	0.1417	0.5182	0.6050
Non-vaccinated	Vaccinated	1	1.1833	4.3285	0.0000
Non-vaccinated	Vaccinated	2	0.9583	3.5055	0.0006
Non-vaccinated	Vaccinated	3	1.2917	4.7248	0.0000
Non-vaccinated	Vaccinated	4	0.5250	1.9204	0.0564
Non-vaccinated	Vaccinated	5	1.6000	4.1384	0.0001
Non-vaccinated	Vaccinated	6	1.4667	3.7936	0.0002
Non-vaccinated	Vaccinated	7	0.4690	1.1559	0.2492
Non-vaccinated	Vaccinated	8	0.4463	0.9404	0.3483
Non-vaccinated	Vaccinated	9	0.5296	1.1160	0.2659
Non-vaccinated	Vaccinated	10	0.3963	0.8350	0.4048
Non-vaccinated	Vaccinated	11	0.3629	0.7648	0.4454
Non-vaccinated	Vaccinated	12	–0.2371	–0.4995	0.6180
Non-vaccinated	Vaccinated	13	0.4629	0.9755	0.3306
Non-vaccinated	Vaccinated	14	0.1629	0.3433	0.7317

The two groups listed in columns A and B were compared.
Orange: Values in columns A are significantly higher than those in column B.

Technical Appendix Table 6. Statistical analyses of bodyweight changes in the Technical Appendix Figure (Comparison of the indicated groups)

A	B	Days post-challenge	Estimate	t-ratio	*P* value
Group 4	Group 2	0	0.0000	0.0000	1.0000
		1	–2.8454	–1.8434	0.0672
		2	–4.7353	–3.0678	0.0025
		3	–7.8214	–5.0671	0.0000
		4	–10.5902	–6.8609	0.0000
		5	–13.9604	–6.3952	0.0000
		6	–17.0433	–7.8075	0.0000
		7	–17.9853	–7.3576	0.0000
		8	–19.9715	–8.1702	0.0000
		9	–19.7374	–6.3707	0.0000
		10	–16.9340	–5.4658	0.0000
		11	–17.7547	–5.7307	0.0000
		12	–15.9514	–5.1487	0.0000
		13	–16.7998	–5.4225	0.0000
		14	–21.7251	–7.0123	0.0000
Group 4	Group 1	0	0.0000	0.0000	1.0000
		1	–1.4601	–0.9459	0.3456
		2	–3.5500	–2.2999	0.0228
		3	–5.8525	–3.7915	0.0002
		4	–7.3417	–4.7563	0.0000
		5	–8.9101	–4.0817	0.0001
		6	–12.1794	–5.5793	0.0000
		7	–12.5117	–5.1184	0.0000
		8	–15.1942	–6.2158	0.0000
		9	–14.7183	–4.7507	0.0000
		10	–12.5474	–4.0500	0.0001
		11	–14.5611	–4.6999	0.0000
		12	–10.6810	–3.4475	0.0007
		13	–12.4662	–4.0237	0.0001
		14	–18.4718	–5.9622	0.0000
Group 4	Group 3	0	0.0000	0.0000	1.0000
		1	0.5914	0.3831	0.7022
		2	–0.6286	–0.4072	0.6844
		3	–0.6640	–0.4302	0.6677
		4	–0.4278	–0.2772	0.7820
		5	–2.5794	–1.1816	0.2392
		6	–2.9783	–1.3643	0.1744
		7	–1.6499	–0.6750	0.5007
		8	–6.1377	–2.2957	0.0230
		9	–9.0080	–2.7447	0.0068
		10	–5.0677	–1.5441	0.1246
		11	–8.9753	–2.7348	0.0070
		12	–4.3746	–1.3329	0.1845
		13	–5.4998	–1.6758	0.0958
		14	–11.3311	–3.4526	0.0007
Group 2	Group 1	0	0.0000	0.0000	1.0000
		1	1.3853	0.8975	0.3709
		2	1.1853	0.7679	0.4437
		3	1.9689	1.2755	0.2040
		4	3.2486	2.1046	0.0369
		5	5.0503	2.3135	0.0220
		6	4.8640	2.2282	0.0273
		7	5.4736	2.5075	0.0132
		8	4.7773	2.1885	0.0301
		9	5.0191	2.2992	0.0228
		10	4.3866	2.0095	0.0462
		11	3.1936	1.4630	0.1455
		12	5.2704	2.4143	0.0169
		13	4.3337	1.9852	0.0489
		14	3.2533	1.4903	0.1382
Group 2	Group 3	0	0.0000	0.0000	1.0000
		1	3.4368	2.2265	0.0274
		2	4.1067	2.6605	0.0086

A	B	Days post-challenge	Estimate	t-ratio	*P* value
		3	7.1574	4.6369	0.0000
		4	10.1624	6.5837	0.0000
		5	11.3811	5.2136	0.0000
		6	14.0650	6.4432	0.0000
		7	16.3354	7.4832	0.0000
		8	13.8338	5.6593	0.0000
		9	10.7294	4.3893	0.0000
		10	11.8663	4.8544	0.0000
		11	8.7794	3.5916	0.0004
		12	11.5768	4.7360	0.0000
		13	11.3001	4.6228	0.0000
		14	10.3940	4.2521	0.0000
Group 1	Group 3	0	0.0000	0.0000	1.0000
		1	2.0515	1.3290	0.1858
		2	2.9215	1.8927	0.0603
		3	5.1885	3.3614	0.0010
		4	6.9138	4.4791	0.0000
		5	6.3307	2.9001	0.0043
		6	9.2011	4.2150	0.0000
		7	10.8617	4.9757	0.0000
		8	9.0565	3.7049	0.0003
		9	5.7103	2.3360	0.0208
		10	7.4798	3.0599	0.0026
		11	5.5858	2.2851	0.0237
		12	6.3065	2.5799	0.0108
		13	6.9664	2.8499	0.0050
		14	7.1407	2.9212	0.0040

The two groups listed in columns A and B were compared.
Orange: Values in columns A are significantly higher than those in column B.
Cyan: Values in columns B are significantly higher than those in column A.

Technical Appendix Table 7. Statistical analyses of bodyweight changes in the Technical Appendix Figure [Comparison of vaccinated (groups 1 and 2 merged) and unvaccinated (groups 3 and 4 merged) groups].

A	B	Days post-challenge	Estimate	t-ratio	*P* value
Non-vaccinated	Vaccinated	0	0.0000	0.0000	1.0000
Non-vaccinated	Vaccinated	1	−2.4485	−2.2821	0.0237
Non-vaccinated	Vaccinated	2	−3.8284	−3.5683	0.0005
Non-vaccinated	Vaccinated	3	−6.5050	−6.0630	0.0000
Non-vaccinated	Vaccinated	4	−8.7520	−8.1574	0.0000
Non-vaccinated	Vaccinated	5	−10.1456	−6.6866	0.0000
Non-vaccinated	Vaccinated	6	−13.1222	−8.6484	0.0000
Non-vaccinated	Vaccinated	7	−13.7356	−8.6156	0.0000
Non-vaccinated	Vaccinated	8	−14.4331	−8.4747	0.0000
Non-vaccinated	Vaccinated	9	−11.5925	−6.1986	0.0000
Non-vaccinated	Vaccinated	10	−11.7323	−6.2734	0.0000
Non-vaccinated	Vaccinated	11	−10.5443	−5.6382	0.0000
Non-vaccinated	Vaccinated	12	−10.7698	−5.7587	0.0000
Non-vaccinated	Vaccinated	13	−11.3365	−6.0617	0.0000
Non-vaccinated	Vaccinated	14	−12.9144	−6.9055	0.0000

The two groups listed in columns A and B were compared.
Cyan: Values in columns B are significantly higher than those in column A.

Technical Appendix Table 8. Statistical analyses of nasal swab titers in Figure 2, panel A (Comparison of the indicated groups)

Group A	Group B	Days post-challenge	Estimate	t-ratio	*P* value
Group 4	Group 2	1	0.9364	2.3933	0.0185
		2	0.4640	1.1860	0.2384
		3	2.9255	7.4770	0.0000
		4	3.2619	8.3367	0.0000
		5	4.7591	8.6007	0.0000
		6	4.7011	8.4959	0.0000
		7	2.7733	4.4827	0.0000
Group 4	Group 1	1	0.3571	0.9128	0.3635
		2	0.0909	0.2324	0.8167
		3	1.8088	4.6230	0.0000
		4	2.3067	5.8954	0.0000
		5	4.7591	8.6007	0.0000
		6	4.7011	8.4959	0.0000

Group A	Group B	Days post-challenge	Estimate	t-ratio	*P* value
		7	2.7733	4.4827	0.0000
Group 4	Group 3	1	–0.0891	–0.2276	0.8204
		2	0.2704	0.6911	0.4910
		3	–0.1784	–0.4560	0.6494
		4	0.1618	0.4135	0.6801
		5	0.0813	0.1470	0.8834
		6	0.3496	0.6318	0.5289
		7	0.8748	1.4140	0.1604
Group 2	Group 1	1	–0.5793	–1.4805	0.1418
		2	–0.3731	–0.9536	0.3425
		3	–1.1167	–2.8540	0.0052
		4	–0.9552	–2.4413	0.0163
		5	0.0000	0.0000	1.0000
		6	0.0000	0.0000	1.0000
		7	0.0000	0.0000	1.0000
Group 2	Group 3	1	–1.0255	–2.6209	0.0101
		2	–0.1936	–0.4949	0.6218
		3	–3.1040	–7.9330	0.0000
		4	–3.1002	–7.9233	0.0000
		5	–4.6778	–8.4537	0.0000
		6	–4.3515	–7.8641	0.0000
		7	–1.8985	–3.4310	0.0009
Group 1	Group 3	1	–0.4462	–1.1404	0.2568
		2	0.1795	0.4587	0.6474
		3	–1.9873	–5.0789	0.0000
		4	–2.1449	–5.4820	0.0000
		5	–4.6778	–8.4537	0.0000
		6	–4.3515	–7.8641	0.0000
		7	–1.8985	–3.4310	0.0009

The two groups listed in columns A and B were compared.
Orange: Values in columns A are significantly higher than those in column B.
Cyan: Values in columns B are significantly higher than those in column A.

Technical Appendix Table 9. Statistical analyses of nasal swab titers in Figure 2, panel A [Comparison of vaccinated (groups 1 and 2 merged) and unvaccinated (groups 3 and 4 merged) groups].

A	B	Days post-challenge	Estimate	t-ratio	*P* value
Non-vaccinated	Vaccinated	1	0.6913	2.5238	0.0131
Non-vaccinated	Vaccinated	2	0.1423	0.5194	0.6045
Non-vaccinated	Vaccinated	3	2.4564	8.9678	0.0000
Non-vaccinated	Vaccinated	4	2.7034	9.8697	0.0000
Non-vaccinated	Vaccinated	5	4.7185	12.1807	0.0000
Non-vaccinated	Vaccinated	6	4.5263	11.6847	0.0000
Non-vaccinated	Vaccinated	7	2.2603	5.5538	0.0000

The two groups listed in columns A and B were compared.
Orange: Values in columns A are significantly higher than those in column B.

Technical Appendix Table 10. Statistical analyses of throat swab titers in Figure 2, panel A (Comparison of the indicated groups)

A	B	Days post-challenge	Estimate	t-ratio	*P* value
Group 4	Group 2	1	1.7086	4.1442	0.0001
		2	1.0755	2.6085	0.0105
		3	3.8785	9.4070	0.0000
		4	4.1299	10.0168	0.0000
		5	5.0419	8.6470	0.0000
		6	4.8938	8.3930	0.0000
		7	4.2523	6.5030	0.0000
Group 4	Group 1	1	0.5150	1.2492	0.2146
		2	0.7191	1.7441	0.0843
		3	2.3366	5.6672	0.0000
		4	2.6004	6.3071	0.0000
		5	5.0419	8.6470	0.0000
		6	4.8938	8.3930	0.0000
		7	4.2523	6.5030	0.0000
Group 4	Group 3	1	0.2125	0.5153	0.6075
		2	–0.1370	–0.3324	0.7403
		3	0.2038	0.4942	0.6223
		4	–0.1453	–0.3525	0.7252

A	B	Days post-challenge	Estimate	t-ratio	*P* value
		5	0.3165	0.5429	0.5885
		6	0.7407	1.2703	0.2070
		7	1.1074	1.6936	0.0935
Group 2	Group 1	1	−1.1936	−2.8950	0.0047
		2	−0.3564	−0.8644	0.3895
		3	−1.5419	−3.7398	0.0003
		4	−1.5295	−3.7097	0.0003
		5	0.0000	0.0000	1.0000
		6	0.0000	0.0000	1.0000
		7	0.0000	0.0000	1.0000
Group 2	Group 3	1	−1.4962	−3.6289	0.0005
		2	−1.2125	−2.9409	0.0041
		3	−3.6747	−8.9128	0.0000
		4	−4.2752	−10.3693	0.0000
		5	−4.7254	−8.1041	0.0000
		6	−4.1531	−7.1227	0.0000
		7	−3.1449	−5.3935	0.0000
Group 1	Group 3	1	−0.3026	−0.7339	0.4648
		2	−0.8561	−2.0765	0.0405
		3	−2.1328	−5.1729	0.0000
		4	−2.7457	−6.6595	0.0000
		5	−4.7254	−8.1041	0.0000
		6	−4.1531	−7.1227	0.0000
		7	−3.1449	−5.3935	0.0000

The two groups listed in columns A and B were compared.
Orange: Values in columns A are significantly higher than those in column B.
Cyan: Values in columns B are significantly higher than those in column A.

Technical Appendix Table 11. Statistical analyses of throat swab titers in Figure 2, panel A [Comparison of vaccinated (groups 1 and 2 merged) and unvaccinated (groups 3 and 4 merged) groups].

A	B	Days post-challenge	Estimate	t-ratio	*P* value
Non-vaccinated	Vaccinated	1	1.0056	3.1990	0.0018
Non-vaccinated	Vaccinated	2	0.9658	3.0723	0.0027
Non-vaccinated	Vaccinated	3	3.0057	9.5613	0.0000
Non-vaccinated	Vaccinated	4	3.4378	10.9361	0.0000
Non-vaccinated	Vaccinated	5	4.8836	10.9852	0.0000
Non-vaccinated	Vaccinated	6	4.5235	10.1750	0.0000
Non-vaccinated	Vaccinated	7	3.5427	7.5786	0.0000

The two groups listed in columns A and B were compared.
Orange: Values in columns A are significantly higher than those in column B.

Technical Appendix Table 12. Statistical analyses of brain titers in Figure 2, panel B (Comparison of the indicated groups)

A	B	LWR	UPR	Difference	Adjusted *P* value
Group 2	Group 4	−3.8181	1.7667	−1.0257	0.6569
Group 1	Group 4	−3.8181	1.7667	−1.0257	0.6569
Group 3	Group 4	−3.1334	2.4514	−0.3410	0.9783
Group 1	Group 2	−2.7924	2.7924	0.0000	1.0000
Group 3	Group 2	−2.1078	3.4770	0.6846	0.8592
Group 3	Group 1	−2.1078	3.4770	0.6846	0.8592

The two groups listed in columns A and B were compared.
LWR: Lower confidence interval
UPR: Upper confidence interval

Technical Appendix Table 13. Statistical analyses of brain titers in Figure 2, panel B [Comparison of vaccinated (groups 1 and 2 merged) and unvaccinated (groups 3 and 4 merged) groups].

A	B	LWR	UPR	Difference	Adjusted *P* value
Vaccinated	Non-vaccinated	−2.0956	0.3853	−0.8551	0.1556

The two groups listed in columns A and B were compared.
LWR: Lower confidence interval
UPR: Upper confidence interval

Technical Appendix Table 14. Statistical analyses of lung titers in Figure 2, panel B (Comparison of the indicated groups)

A	B	LWR	UPR	Difference	Adjusted *P* value
Group 2	Group 4	−7.3317	−3.7885	−5.5601	0.0000
Group 1	Group 4	−7.3317	−3.7885	−5.5601	0.0000
Group 3	Group 4	−1.3220	2.2212	0.4496	0.8469
Group 1	Group 2	−1.7716	1.7716	0.0000	1.0000
Group 3	Group 2	4.2381	7.7813	6.0097	0.0000

Group 3	Group 1	4.2381	7.7813	6.0097	0.0000

The two groups listed in columns A and B were compared.
Orange: Values in columns A are significantly higher than those in column B.
Cyan: Values in columns B are significantly higher than those in column A.
LWR: Lower confidence interval
UPR: Upper confidence interval

Technical Appendix Table 15. Statistical analyses of lung titers in Figure 2, panel B [Comparison of vaccinated (groups 1 and 2 merged) and unvaccinated (groups 3 and 4 merged) groups].

A	B	LWR	UPR	Difference	Adjusted *P* value
Vaccinated	Non-vaccinated	–6.5960	–4.9737	–5.7849	0.0000

The two groups listed in columns A and B were compared.
Cyan: Values in columns B are significantly higher than those in column A.
LWR: Lower confidence interval
UPR: Upper confidence interval

Technical Appendix Table 16. Statistical analyses of nasal turbinate titers in Figure 2, panel B (Comparison of the indicated groups)

A	B	LWR	UPR	Difference	Adjusted *P* value
Group 2	Group 4	–11.8235	2.0552	–4.8841	0.1885
Group 1	Group 4	–9.8570	4.0217	–2.9177	0.5621
Group 3	Group 4	–7.0473	6.8314	–0.1079	1.0000
Group 1	Group 2	–4.9729	8.9058	1.9664	0.8017
Group 3	Group 2	–2.1632	11.7155	4.7762	0.2017
Group 3	Group 1	–4.1296	9.7491	2.8097	0.5896

The two groups listed in columns A and B were compared.
LWR: Lower confidence interval
UPR: Upper confidence interval

Technical Appendix Table 17. Statistical analyses of nasal turbinate titers in Figure 2, panel B [Comparison of vaccinated (groups 1 and 2 merged) and unvaccinated (groups 3 and 4 merged) groups].

A	B	LWR	UPR	Difference	Adjusted *P* value
Vaccinated	Non-vaccinated	–7.0544	–0.6395	–3.8469	0.0234

The two groups listed in columns A and B were compared.
Cyan: Values in columns B are significantly higher than those in column A.
LWR: Lower confidence interval
UPR: Upper confidence interval

Technical Appendix Table 18. Statistical analyses of olfactory bulb titers in Figure 2, panel B (Comparison of the indicated groups)

A	B	LWR	UPR	Difference	Adjusted *P* value
Group 2	Group 4	–2.5603	3.1929	0.3163	0.9839
Group 1	Group 4	–2.8465	2.9067	0.0301	1.0000
Group 3	Group 4	0.0017	5.7549	2.8783	0.0499
Group 1	Group 2	–3.1628	2.5904	–0.2862	0.9880
Group 3	Group 2	–0.3146	5.4386	2.5620	0.0820
Group 3	Group 1	–0.0284	5.7248	2.8482	0.0523

The two groups listed in columns A and B were compared.
Orange: Values in columns A are significantly higher than those in column B.
LWR: Lower confidence interval
UPR: Upper confidence interval

Technical Appendix Table 19. Statistical analyses of olfactory bulb titers in Figure 2, panel B [Comparison of vaccinated (groups 1 and 2 merged) and unvaccinated (groups 3 and 4 merged) groups].

A	B	LWR	UPR	Difference	Adjusted *P* value
Vaccinated	Non-vaccinated	–3.1841	0.6521	–1.2660	0.1722

The two groups listed in columns A and B were compared.
LWR: Lower confidence interval
UPR: Upper confidence interval

Technical Appendix Table 20. Statistical analyses of tracheal titers in Figure 2, panel B (Comparison of the indicated groups)

A	B	LWR	UPR	Difference	Adjusted *P* value
Group 2	Group 4	–8.1933	–5.3690	–6.7811	0.0000
Group 1	Group 4	–8.1933	–5.3690	–6.7811	0.0000
Group 3	Group 4	–1.7448	1.0795	–0.3326	0.8724
Group 1	Group 2	–1.4121	1.4121	0.0000	1.0000
Group 3	Group 2	5.0363	7.8606	6.4485	0.0000
Group 3	Group 1	5.0363	7.8606	6.4485	0.0000

The two groups listed in columns A and B were compared.
Orange: Values in columns A are significantly higher than those in column B.
Cyan: Values in columns B are significantly higher than those in column A.
LWR: Lower confidence interval

A	B	LWR	UPR	Difference	Adjusted *P* value

UPR: Upper confidence interval

Technical Appendix Table 21. Statistical analyses of tracheal titers in Figure 2, panel B [Comparison of vaccinated (groups 1 and 2 merged) and unvaccinated (groups 3 and 4 merged) groups].

A	B	LWR	UPR	Difference	Adjusted *P* value
Vaccinated	Non-vaccinated	–7.2579	–5.9717	–6.6148	0.0000

The two groups listed in columns A and B were compared.
Cyan: Values in columns B are significantly higher than those in column A.
LWR: Lower confidence interval
UPR: Upper confidence interval

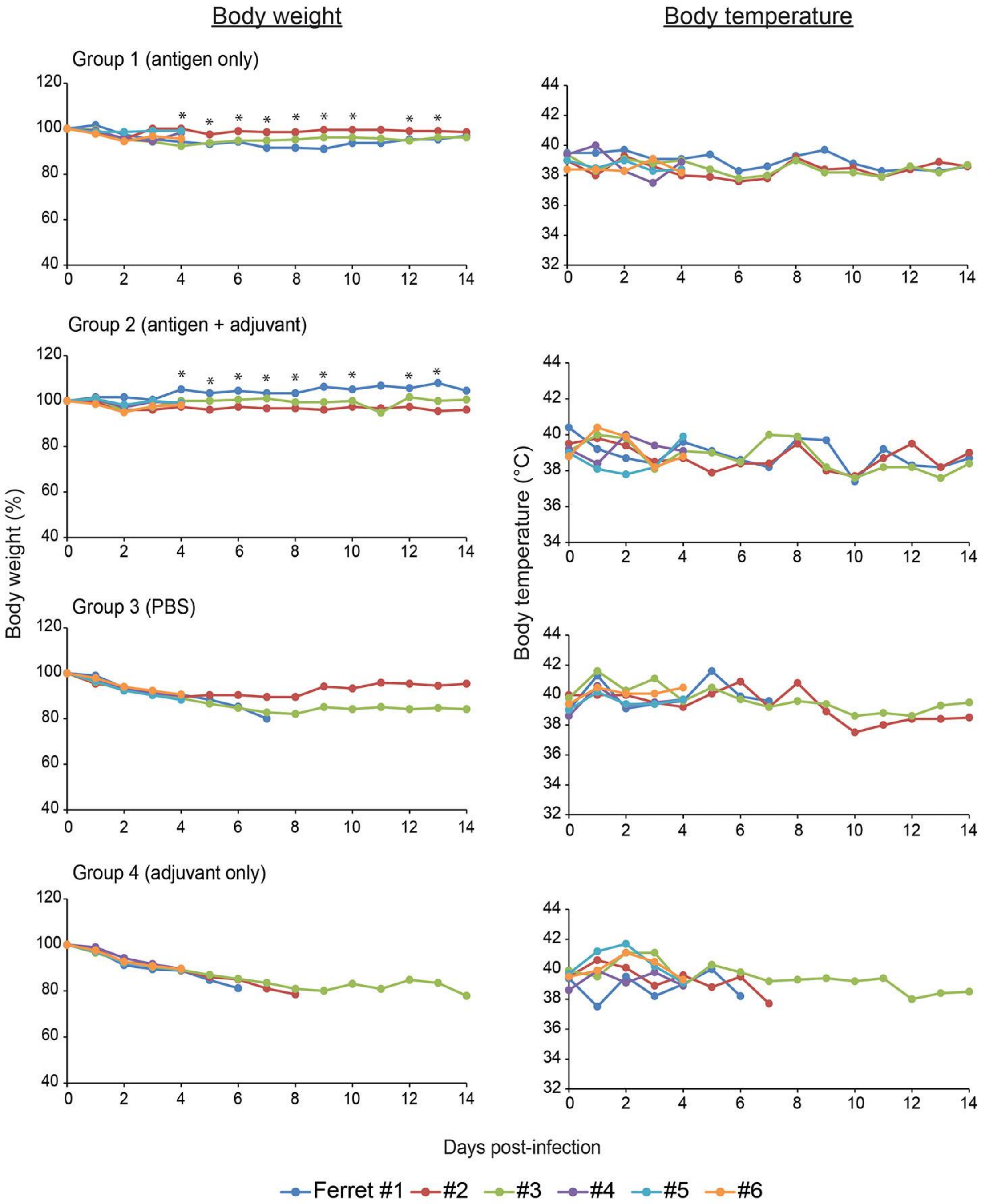

Technical Appendix Figure. Bodyweight and temperature changes in vaccinated and non-vaccinated ferrets challenged with highly pathogenic H7N9 virus. Six ferrets per group were challenged intranasally with 10^6 PFU of highly pathogenic H7N9 rGD/3-NA294R virus; bodyweight and temperature were monitored daily for 14 days. Ferrets #4 – #6 in each group were euthanized on day 4 post-challenge for organ sampling. Ferret #1 in group 3, and ferrets #1 and #2 in group 4 were euthanized on days 7, 6, and 8 post-challenge, respectively, due to severe symptoms. Statistically significant differences in bodyweight changes between ferrets in Groups 1 and 2 are marked (*); *, $p \leq 0.05$.
